# Calmodulin regulates the olfactory performance in *Drosophila melanogaster*

**DOI:** 10.1038/s41598-021-83296-9

**Published:** 2021-02-12

**Authors:** Kalpana Jain, Sofia Lavista-Llanos, Veit Grabe, Bill S. Hansson, Dieter Wicher

**Affiliations:** Department of Evolutionary Neuroethology, Max Planck Institute for Chemical Ecology, Hans-Knöll-Strasse 8, 07745 Jena, Germany

**Keywords:** Cell biology, Neuroscience, Physiology

## Abstract

Insect odorant receptors (ORs) detect volatile chemical cues with high sensitivity. These ORs operate as ligand-gated ion channels and are formed by heptahelical OrX and Orco (co-receptor) proteins. A highly conserved calmodulin (CaM) binding site (CBS) ^336^SAIKYWVER^344^ within the second intracellular loop of *Drosophila melanogaster* Orco constitutes a target for regulating OR performance. Here we asked how a point mutation K339N in this CBS affects the olfactory performance of *Drosophila melanogaster*. We first asked how this mutation would affect the odor responses of olfactory sensory neurons (OSNs). Using Ca^2+^ imaging in an ex-vivo antenna preparation, we activated all OR (OrX/Orco) expressing neurons using the synthetic agonist VUAA1. In a next attempt, we restricted the OR spectrum to Or22a expressing neurons (Or22a/Orco) and stimulated these OSNs with the ligand ethyl hexanoate. In both approaches, we found that flies carrying the K339N point mutation in Orco display a reduced olfactory response. We also found that the mutation abolishes the capability of OSNs to sensitize by repeated weak odor stimuli. Next, we asked whether OrcoK339N might affect the odor localization performance. Using a wind tunnel bioassay, we found that odor localization in flies carrying the OrcoK339N mutation was severely diminished.

## Introduction

For insects, the sense of smell plays a significant role in detecting and distinguishing between different types of odors present in nature. This function is vital for finding mating partners, food sources and oviposition sites, as well as to avoid harmful sites indicated by alarm signals^1,2^. Using their olfactory organs, the antennae and the maxillary palps, insects detect such odors over short as well as long distances^3–6^. The 3^rd^ antennal segment is equipped with sensory hair-like structures, sensilla, which contain the dendrites of olfactory sensory neurons (OSNs), where three different types of olfactory receptors are localized^4^. With these receptors, the OSNs convert information about the odor chemical properties into electrochemical signals^7,8^. The present study focuses on odorant receptors (ORs). OR proteins display a seven-transmembrane topology with intracellular N-terminus and extracellular C-terminus^9–12^. They form heteromeric assemblies of variable odor-specific OrX proteins and a conserved co-receptor protein, Orco^9,13–16^. ORs operate as odor-activated non-selective cation channels permeable to Na^+^, K^+^, and Ca^2+^^15,17^. Heterologously expressed Orco proteins also form non-selective cation channels, not activated by odors, but by cyclic nucleotides^15^ or by synthetic agonists such as VUAA1^18,19^. While structural information of ORs is still lacking, the structure of an Orco channel from the wasp *Apocrypta bakeri* was recently accomplished by cryo-electron microscopy^10^. Similar to voltage-gated channels, Orco channels are tetrameric assemblies with a central ion-conducting pore.

With a given primary function as ligand-gated channels, the functional properties of ORs are—as known for other channels and receptors—subject to regulation by a variety of mechanisms^20,21^. The ligand sensitivity of ORs, for example, is regulated by intracellular signaling cascades including players such as phospholipase C (PLC), protein kinase C (PKC) and 3′,5′-cyclic adenosine monophosphate (cAMP)^22–25^. The function of many of these regulators depends on the intracellular Ca^2+^ level that is, among others, affected by the OR activity. This functional architecture gives rise to feedback loops.

Orco is target of many regulatory processes. Electrophysiological and behavioral studies have shown that PLC, PKC, and adenylyl cyclase (AC) activity modulate the function of Orco, which in turn affects the OR performance^22,24^. Flies carrying mutated PKC phosphorylation sites in Orco, or expressing deficient PKC genes, show a reduced olfactory performance and a disturbed capability for odor localization^25^. Moreover, prolonged odor exposure leads to Orco de-phosphorylation, which results in reduced odorant sensitivity and affects the behavior of the fly^26^.

Subthreshold odor stimulation, when repeated within a suitable time window, leads to OR sensitization^24^. The process of OR sensitization relies on optimal Orco function. In flies expressing Orco with mutated PKC phosphorylation sites, or in those with pharmacologically downregulated PKC or AC function, no sensitization could be observed^24^.

An important regulator of cellular function is calmodulin (CaM), a Ca^2+^ sensor that binds to a multitude of target proteins including ion channels and transporters^27–31^. In a couple of in-vitro and ex-vivo studies CaM has been demonstrated to regulate the function of insect ORs via targeting Orco^32–35^. Insect Orco proteins possess a conserved CaM binding site (CBS) ^336^SAIKYWVER^344^^32–36^. In the presence of the CaM inhibitor W7, heterologously expressed Orco homomers show reduced Ca^2+^ responses when stimulated with VUAA1^32,34^. Similarly, reduced responses were also observed with Orco constructs bearing the point mutation K339N within the CBS^32^. Furthermore, with disrupted CaM action, either by pharmacological CaM inhibition or with point mutated Orco CBS, no OR sensitization could be elicited^33^. In a previous study, more severe modifications within the Orco CBS, i.e. the deletion of the N-terminal and the C-terminal part of the CBS, the deletion of W341 as well as the substitution of two positive by two negative charges RH344EE were found to prevent the traffic of Orco and OrX proteins to the outer dendrites^35^.

The present work was aimed to investigate the effects of the point mutation K339N in the Orco CBS—that does not disrupt OR trafficking to the dendritic membrane—on the olfactory performance of the fly. Thereby we expand the investigation performed with heterologously expressed OR proteins^32^ to the level of OSNs in their native environment, and to the behavioral performance of the fly. Initially, we performed Ca^2+^ imaging experiments in an ex-vivo antenna preparation, as previously developed^32^. Next, we tested the effect that the K339N CBS mutation of Orco has on OSN sensitization. We asked whether the K339N mutation would affect the performance of the fly in locating an odor source in a wind tunnel bioassay.

## Results

### Point mutation in Orco CBS affects the OSN odor response

**Figure 1:**
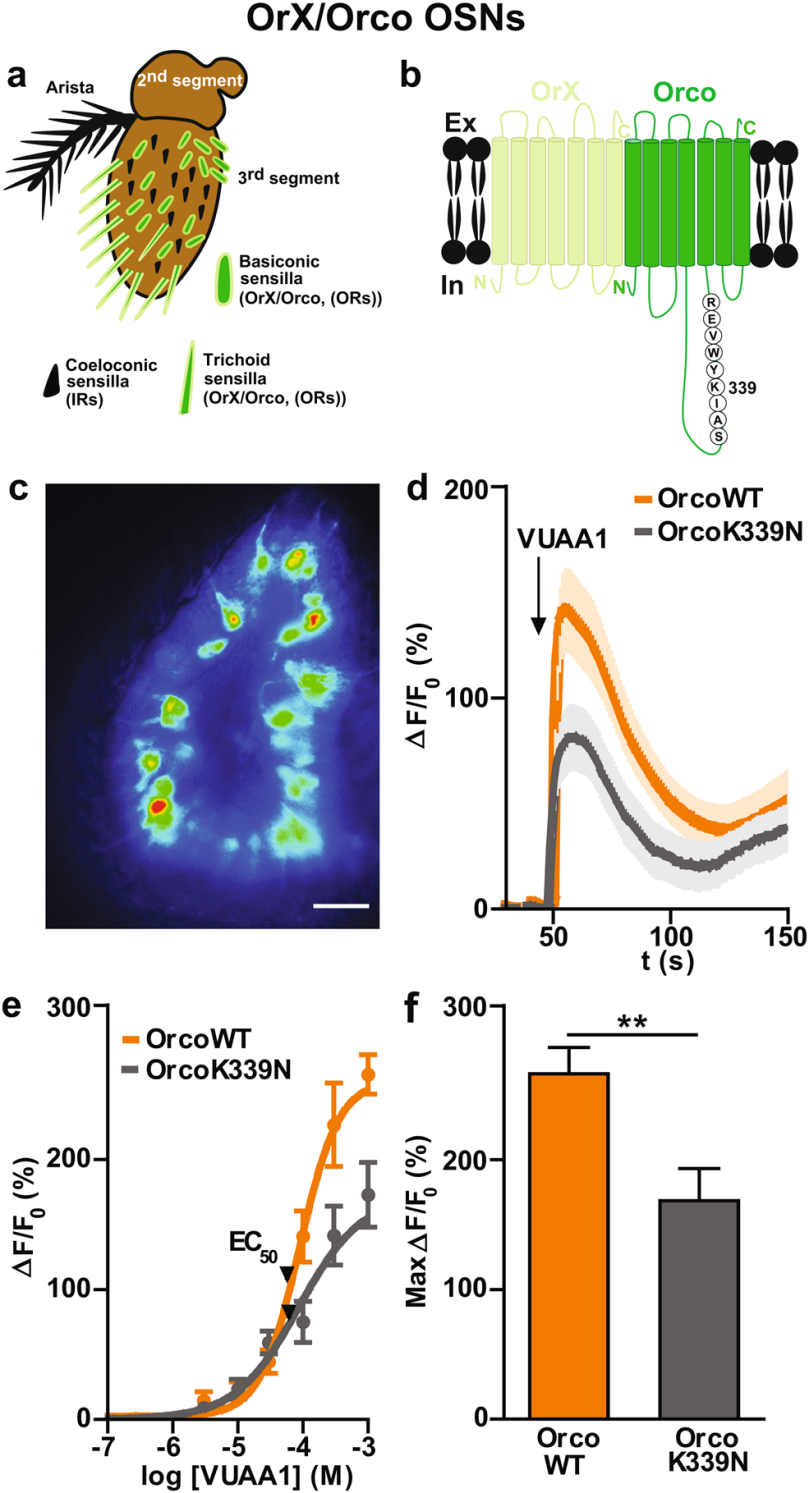
Effect of Orco CBS mutation on the odor response in OrX/Orco expressing OSNs. **(a)** Scheme of a *Drosophila melanogaster* antenna. The third segment is covered with sensilla that house the dendrites of up to four OSNs. OSNs that express ORs (odorant receptor) are present in basiconic and trichoid sensilla and coeloconic sensilla house IR expressing OSNs. **(b)** Schematic view of insect ORs formed by heptahelical OrX and Orco (co-receptor) proteins. The second intracellular loop of Orco contains the highly conserved CaM binding site ^336^SAIKYWVER^344^. **(c)** GCaMP labelled fluorescence intensity of OrX/Orco expressing OSNs. **(d)** Time course of Ca^2+^ fluorescence responses obtained in OrcoWT OSNs and OrcoK339N OSNs upon ORs stimulation with 100 µM VUAA1 (n = 10 for OrcoWT, n = 11 for OrcoK339N). **(e)** Concentration response relationship for OrcoWT and OrcoK339N (7 ≤ n ≤ 11). Curves represent sigmoidal fits described by Hill coefficient 1.44 (OrcoWT) and 0.89 (OrcoK339N), and EC_50_ of 88 µM (OrcoWT), 85 µM (OrcoK339N). **(f)** Maximum fluorescence response (Max ∆F/F_0_ (%)) obtained by subtracting the base level at 50 s from the maximum Ca^2+^ fluorescence at 1 mM VUAA1. n = number of antennae. Two-tailed unpaired *t-* test*,* **P < 0.01. Data given as mean ± SEM. Scale bar of antenna: 20 µm.

We first generated flies that express a mutated version of Orco carrying the point mutation K339N (OrX/OrcoK339N) along with the calcium indicator GCaMP6f, in all OR expressing OSNs (OrX/Orco OSNs) (Fig. 1a, b). Then we checked by immunohistochemistry whether the mutation affects OR trafficking to the OSN dendrites, and compared the expression of Or22a in mutant flies with UAS-OrcoK339N, as well as in Orco wild type expressing OSNs (OrX/OrcoWT) and in *Orco*^*-/-*^ mutant flies rescued with a wild type Orco (OrX/OrcoWT-R) control lines. These tests revealed that the Or22a protein was normally expressed in the outer dendrites of OrX/OrcoK339N OSNs (Supplementary Fig. S1).

We monitored the OR activity by Ca^2+^ imaging experiments using an ex vivo* Drosophila* antenna preparation according to our previous study^32^. This preparation allows to observe the OR expressing OSNs within their native environment (Fig. 1c). OR stimulation with the synthetic Orco agonist VUAA1 initiated a Ca^2+^ influx that lead to a rise in [Ca^2+^]_i_ visualized as an increase in GCaMP6f fluorescence intensity. We compared the change in fluorescence intensity ∆F/F_0_ upon OR stimulation of OrX/OrcoWT OSNs to that of OSNs expressing OrX/OrcoK339N (Fig. 1d). In both cases, OR stimulation with 100 µM VUAA1 elicited a fast and strong rise in Ca^2+^ fluorescence. After reaching a peak within a few seconds (6.7 ± 2.1 s for OrX/OrcoWT (n = 10) and 7.6 ± 2.3 s for OrX/OrcoK339N (n = 11); ns, not significant) the maximum change in fluorescence intensity declined mono-exponentially (time constant τ = 69 ± 7 s for OrX/OrcoWT and 41 ± 8 s for OrX/OrcoK339N; *P < 0.05). With a Max ∆F/F_0_ of 138 ± 19% for OrX/OrcoWT and 78 ± 16% for OrX/OrcoK339N (*P < 0.05), the odor response of OrX/OrcoK339N OSNs was clearly weaker and shorter than that of OrX/OrcoWT (Fig. 1f), at 1 mM concentration. In line with this, the VUAA1 concentration dependence of the fluorescence peak (Fig. 1e, f) showed a reduced efficacy in OrX/OrcoK339N OSNs. Interestingly, the dose–response curves were characterized by comparable EC_50_ in both cases (Fig. 1e).

Next, we asked whether the effects on OR activation seen in OrX/OrcoK339N OSNs would be comparable to the effect of inhibiting the CaM function in OrX/OrcoWT OSNs. For this sake, we pre-incubated the antenna of OrX/OrcoWT with the CaM inhibitor W7^32^ and stimulated the preparation with VUAA1. As shown in Supplementary Fig. S2a, the maximum Ca^2+^ fluorescence response of OrX/OrcoWT OSNs in the presence of W7 was reduced as observed in OrX/OrcoK339N expressing OSNs. Thus, the inhibition of the CaM function affects the odor response of OSNs in a similar manner as mutating the CBS within the Orco protein. However, the effects are not identical as, for example, in the presence of W7 the decay of the OrX/OrcoWT OSN response with 100 µM VUAA1 stimulation was prolonged (τ = 106 ± 8 s, n = 8) rather than reduced.

**Figure 2:**
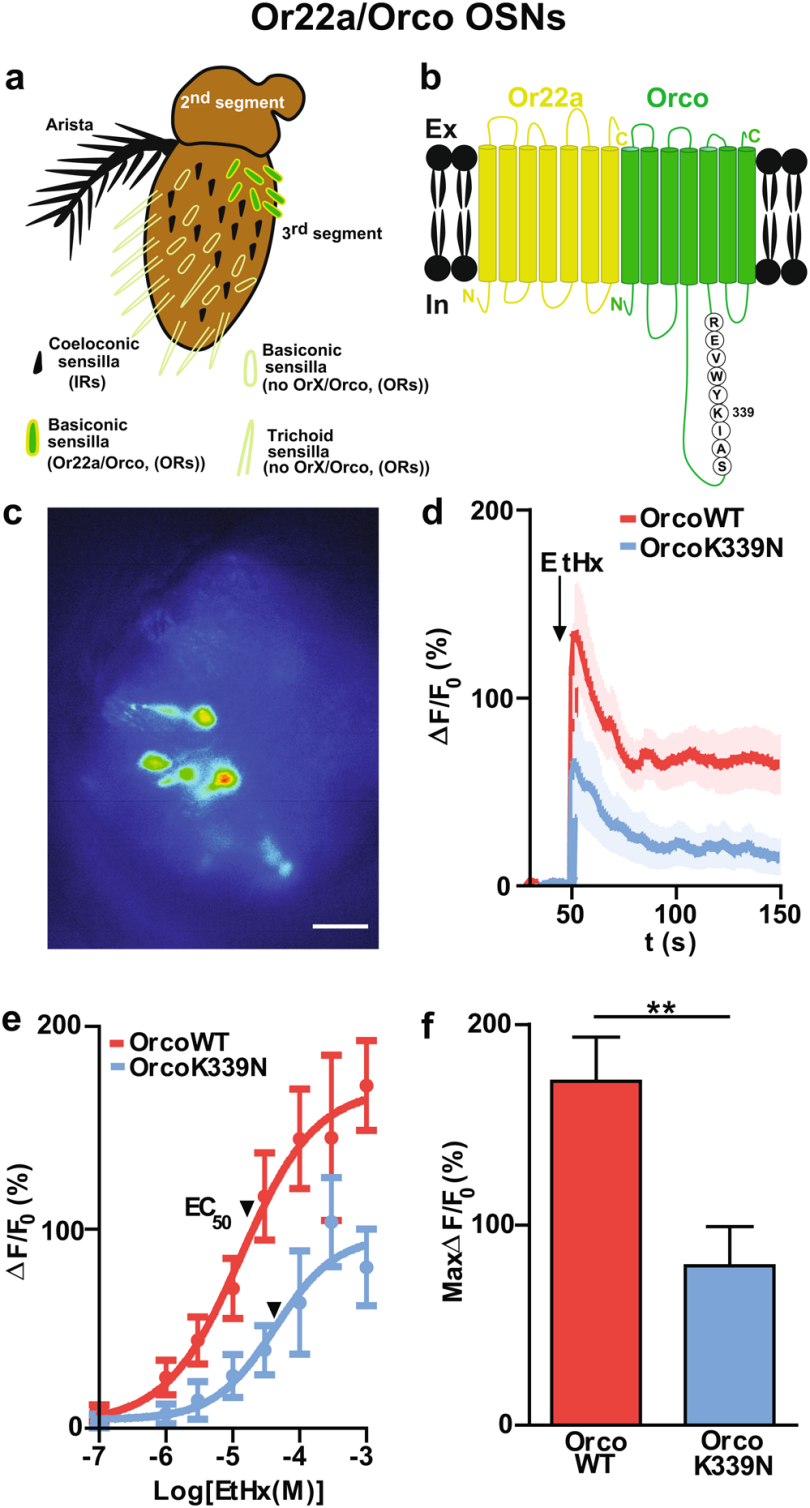
Effect of Orco CBS mutation on the odor response in solely Or22a/Orco expressing OSNs. **(a)** Scheme of a *Drosophila melanogaster* antenna. The third segment is covered with sensilla that house the dendrites of up to four OSNs. OSNs that express ORs (odorant receptor) are present in basiconic and trichoid sensilla and coeloconic sensilla house IR expressing OSNs. **(b)** Schematic view of insect ORs formed by heptahelical Or22a and Orco (co-receptor) proteins. The second intracellular loop of Orco contains the highly conserved CaM binding site ^336^SAIKYWVER^344^. **(c)** GCaMP labelled fluorescence intensity of Or22a/Orco OSNs. **(d)** Time course of Ca^2+^ fluorescence responses obtained OrcoWT OSNs and OrcoK339N OSNs upon OR stimulation with 100 µM ethyl hexanoate (n = 11 for OrcoWT, n = 11 for OrcoK339N). **(e)** Concentration response relationship for OrcoWT and OrcoK339N (8 ≤ n ≤ 10). Curves represent sigmoidal fits described by Hill coefficient 0.777 (OrcoWT), 0.9525 (OrcoK339N), and EC_50_ of 13 µM (OrcoWT), 42 µM (OrcoK339N). **(f)** Maximum fluorescence response (Max ∆F/F_0_ (%)) obtained by subtracting the base level at 50 s from the maximum Ca^2+^ fluorescence at 1 mM ethyl hexanoate. n = number of antennae. Two-tailed unpaired *t-* test*,* **P < 0.01. Data given as mean ± SEM. Scale bar of antenna: 20 µm.

So far, the ORs were activated with the synthetic Orco-specific agonist VUAA1. We next tested how the OrcoK339N mutation would affect the response to a natural OrX-specific ligand. For that, we specifically targeted a single population of OSNs that express the well-characterized and broadly tuned Or22a which is activated by fruit odors like ethyl hexanoate^37^ (Fig. 2a, b). We compared the time course of Ca^2+^ fluorescence responses to 100 μM ethyl hexanoate in Or22a/Orco OSNs expressing either Orco wild type (Or22a/OrcoWT) or K339N point mutated Orco (Or22a/OrcoK339N) (Fig. 2c, d). These flies express only a single functional type of OSN (Or22a) and are, otherwise, *Orco*^*1*^ null mutants (all non-Or22a OSNs express no Orco). The maximum Ca^2+^ fluorescence was obtained at 3.2 ± 1.0 s for Or22a/OrcoWT (n = 11) and 2.7 ± 0.8 s for Or22a/OrcoK339N (n = 11) (ns), with a maximum change in the fluorescence intensity (Max ∆F/F_0_) of 127 ± 23% for Or22a/OrcoWT and 56 ± 22% for Or22a/OrcoK339N, which was statistically significant (*P < 0.05). The decay of the fluorescence signal was a superposition of a fast and a slow process (Fig. 2d). The fast decay could be described by τ = 58 ± 18 s for Or22a/OrcoWT and 99 ± 37 s for Or22a/OrcoK339N. The ethyl hexanoate concentration dependence of the fluorescence peak (Fig. 2e) differed in efficacy and in the (EC_50_) between Or22a/OrcoWT and Or22a/OrcoK339N. Thus, in comparison with Or22a/OrcoWT OSNs, the Or22a/OrcoK339N OSNs showed both a reduced odor response at saturation (1 mM) (Fig. 2f) and a decreased sensitivity to ethyl hexanoate (Fig. 2e).

**Figure 3:**
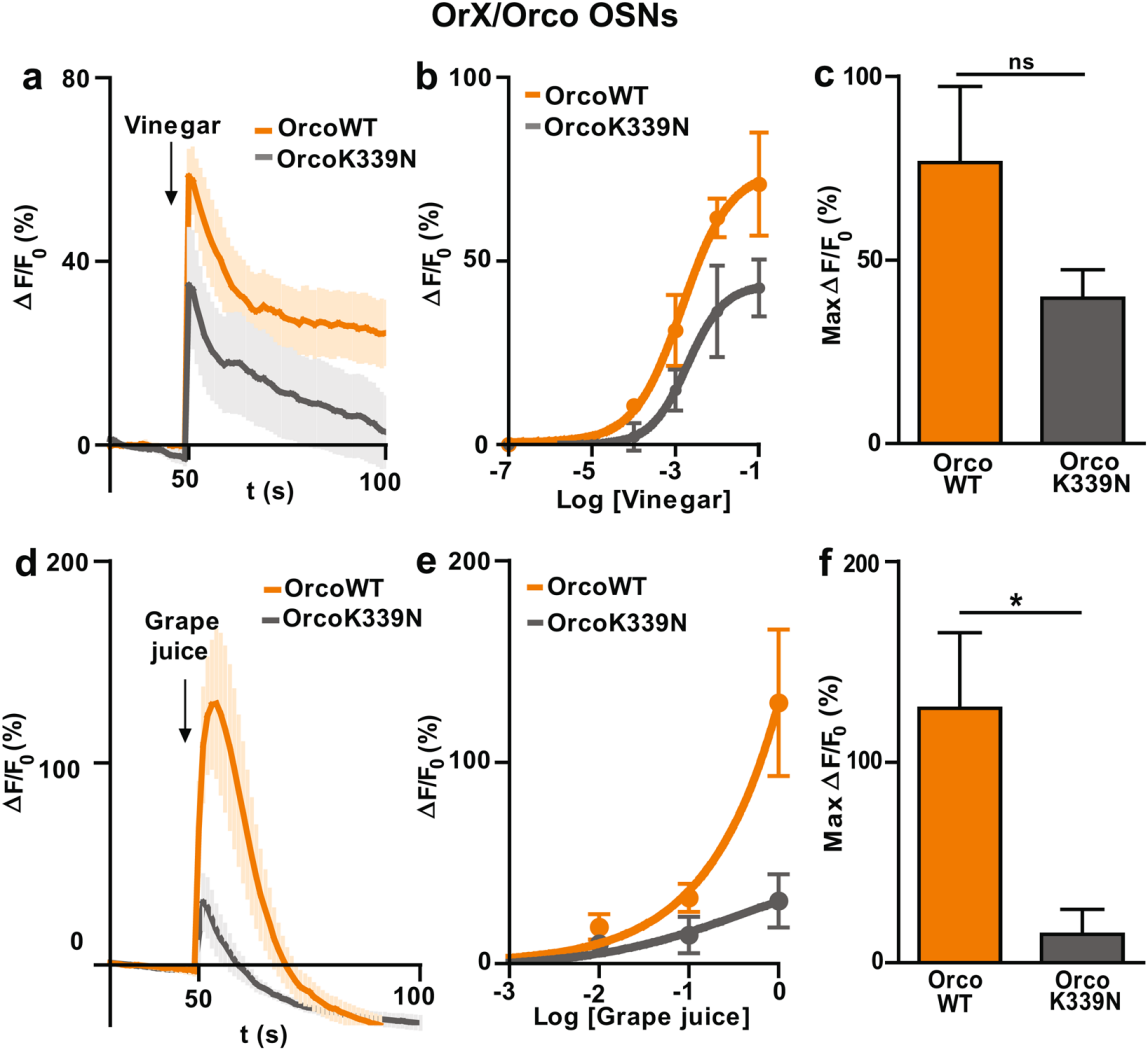
Effect of Orco CBS mutation on the odor response in OrX/Orco expressing OSNs using vinegar and grape juice as stimulus. **(a)** Time course of Ca^2+^ fluorescence responses obtained in OrX/OrcoWT OSNs and OrX/OrcoK339N OSNs upon OR stimulation with 10^–2^ concentration of vinegar (n = 5 for OrcoWT, n = 5 for OrcoK339N). **(b)** Concentration response relationship for OrcoWT and OrcoK339N OSNs (n = 5). **(c)** Maximum Ca^2+^ fluorescence response (Max ∆F/F_0_ (%)) obtained by subtracting the base level at 50 s from the maximum Ca^2+^ fluorescence at 10^–1^ dilution. **(d)** Time course of Ca^2+^ fluorescence responses obtained in OrcoWT and OrcoK339N flies upon OR stimulation with pure grape juice (n = 5 for OrcoWT, n = 5 for OrcoK339N). **(e)** Concentration response relationship for OrcoWT and OrcoK339N OSNs (n = 5). **(f)** Maximum Ca^2+^ fluorescence response (Max ∆F/F_0_ (%)) obtained by subtracting the base level at 50 s from the maximum Ca^2+^ fluorescence obtained for pure grape juice. n = number of antennae. Two-tailed unpaired *t-* test*,* *P < 0.05, ns = not significant. Data given as mean ± SEM.

We next tested the effect of ecologically relevant stimuli (i.e. vinegar and grape juice) on the calcium dynamics of OrX/Orco expressing OSNs. Using vinegar at 10^–2^ dilution (Fig. 3a), we observed an increase in the Ca^2+^ fluorescence response in both OrX/OrcoWT and OrX/OrcoK339N OSNs conditions. The maximum change in the fluorescence intensity (Max ∆F/F_0_) was 57.1 ± 5.4% for OrX/OrcoWT (n = 5) and 32.2 ± 12.8% for OrX/OrcoK339N (n = 5) (ns). The fluorescence peak was reached within a few seconds (2.0 ± 0.7 s (n = 5) for OrX/OrcoWT and 1.2 ± 0.2 s for OrX/OrcoK339N (n = 5); ns), subsequently the fluorescence intensity declined mono-exponentially (τ = 153 ± 23 s for OrX/OrcoWT and 246 ± 57 s for OrX/OrcoK339N; ns). The fluorescence response of OrX/OrcoK339N OSNs was not significantly different to that of OrX/OrcoWT at saturation concentration (10^–1^) (Fig. 3b). Plotting the dose response curve for OrX/OrcoWT and OrX/OrcoK339N at different vinegar dilutions (Fig. 3c) revealed a reduced odor response in OrX/OrcoK339N OSNs compared to OrX/OrcoWT OSNs.

In a following set of experiments, we tested the Ca^2+^ fluorescence response to grape juice, at different dilutions, in all OR expressing neurons. In both OrX/OrcoWT and OrX/OrcoK339N antenna there was a rise in the Ca^2+^ fluorescence response upon stimulation with pure grape juice, as shown in (Fig. 3d, e), with a Max ∆F/F_0_ of 126.9 ± 37.6% in OrX/OrcoWT and 14.2 ± 12.4% in OrX/OrcoK339N (*P < 0.05; n = 5). The Ca^2+^ fluorescence peak was reached within a few seconds (3.6 ± 0.87 s for OrcoWT (n = 5) and 2.0 ± 0.3 s for OrX/OrcoK339N (n = 5); ns), and the decay was delayed in OrX/OrcoK339N OSNs compared to OrX/OrcoWT OSNs (τ = 55 ± 3 s for OrX/OrcoWT and 82 ± 4 s for OrX/OrcoK339N; ***P < 0.001, n = 5) (Fig. 3d). Compared to OrX/OrcoWT, the Ca^2+^ fluorescence response of OrX/OrcoK339N OSNs was shorter at the saturation level (pure grape juice) (Fig. 3e), and comparatively lower at the different grape juice dilutions, as it is shown in dose response curve (Fig. 3f).

### Point mutation in Orco CBS affects OSN sensitization

**Figure 4:**
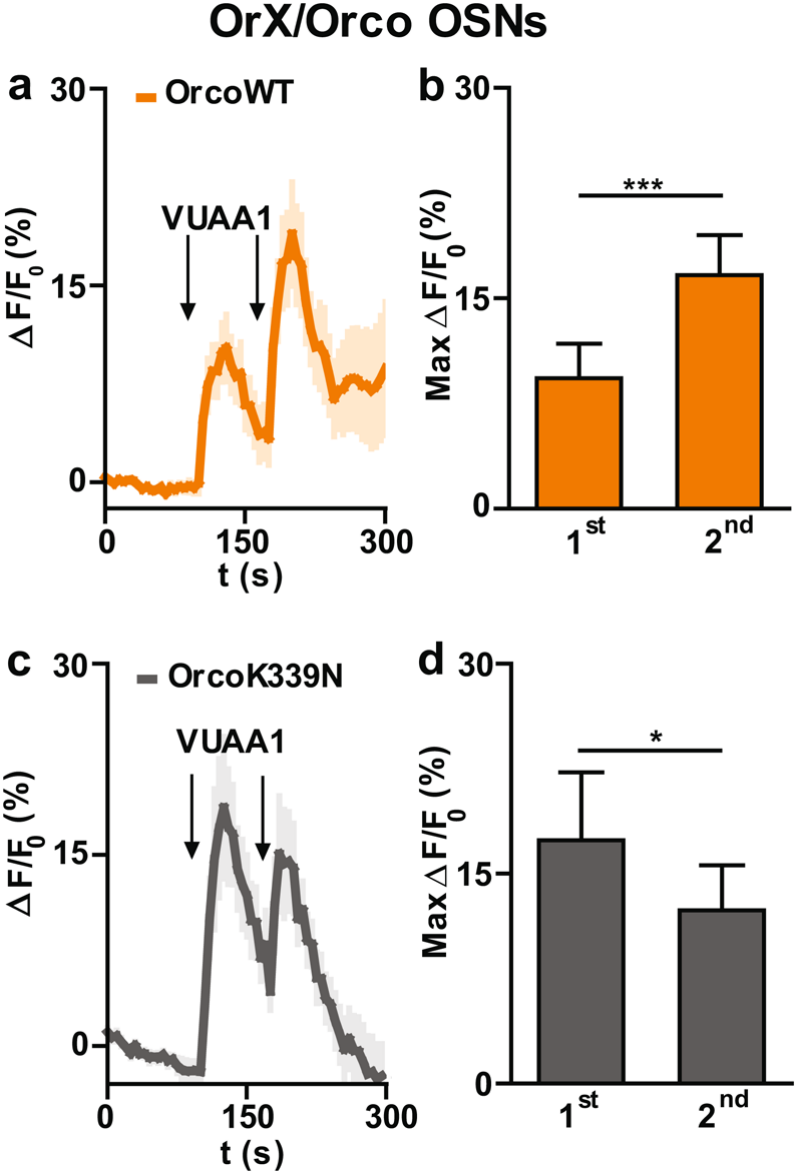
Effect of Orco CBS mutation on the sensitization in OrX/Orco expressing OSNs. (**a,c**) Time course of Ca^2+^ fluorescence response (∆F/F_0_ (%)) in OrX/OrcoWT **(a)** and OrX/OrcoK339N **(c)** OSNs upon ORs stimulation with 3 µM VUAA1 stimulation (n = 16 for OrcoWT, and n = 9 for OrcoK339N). **(b**, **d)** intensity of 1st (first) and 2nd (second) Ca^2+^ fluorescence responses (∆F/F_0_ (%)) for OrcoWT **(b)** and OrcoK339N **(d)**. Intensity of 1st and 2nd response was calculated by subtracting the base level from the maximum Ca^2+^ fluorescence. In case of sensitization, the 2nd response is significantly higher than 1st response. n = cell bodies. Two-tailed paired *t-*test, *P < 0.05, ***P < 0.001. Data given as mean ± SEM.

**Figure 5:**
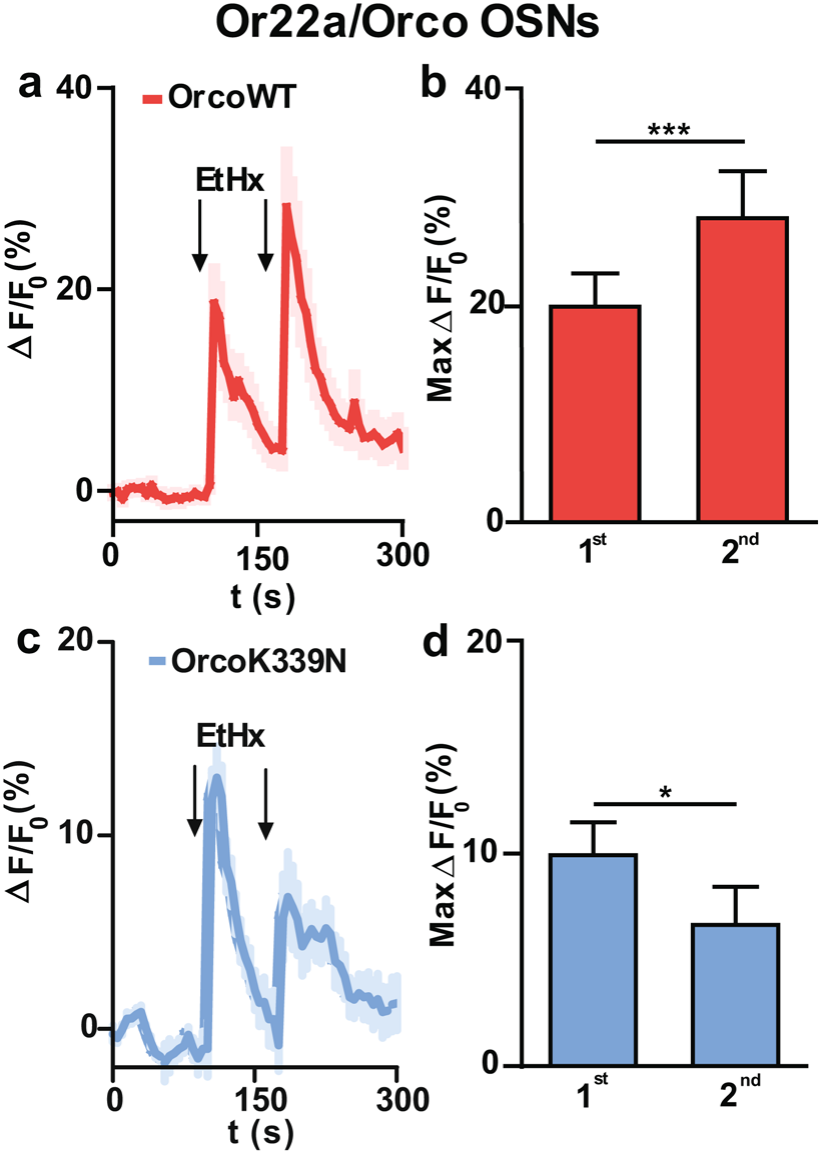
Effect of Orco CBS mutation on the sensitization in Or22a/Orco expressing OSNs. **(a)** Time course of Ca^2+^ fluorescence response (∆F/F_0_ (%)) in OrcoWT OSNs upon OR stimulation with 0.05 µM ethyl hexanoate (n = 14). **(b)** Intensity of 1st and 2nd Ca^2+^ fluorescence responses (∆F/F_0_ (%)) for OrcoWT OSNs. **(c)** Time course of Ca^2+^ fluorescence response (∆F/F_0_ (%)) in OrcoK339N OSNs upon OR stimulation with 0.5 µM ethyl hexanoate (n = 12). **(d)** Intensity of 1st and 2nd Ca^2+^ fluorescence responses (∆F/F_0_ (%)) for OrcoK339N OSNs. Intensity of 1st and 2nd response was calculated by subtracting the base level from the maximum Ca^2+^fluorescence. In case of sensitization, the 2nd response is significantly higher than 1st response. n = cell bodies. Two-tailed paired *t-*test, *P < 0.05, ***P < 0.001. Data given as mean ± SEM.

Previous investigations have shown that Orco activation is necessary for insect OR sensitization^24,33^ and that inhibition of CaM by W7 abolishes this phenomenon in heterologously expressed ORs^33^. We thus asked whether the Orco CBS point mutation K339N may also affect the sensitization phenomenon. In a first step, we established a sensitization protocol in our ex vivo* Drosophila* antenna preparation using two consecutive threshold VUAA1 stimulations. As sensitization is known to occur with stimuli spaced between 10 s to 3 min^24^ we chose a time span of 75 s, and a subthreshold odor concentration^24^ based on our dose response curve (Fig. 1e). With 3 µM VUAA1 we observed sensitization in OrX/OrcoWT OSNs (Fig. 4a, b), but we never observed sensitization in OrX/OrcoK339N OSNs (Fig. 4c, d). Next, we tested the sensitization phenomenon specifically in Or22a OSNs using a threshold concentration of ethyl hexanoate taken from the dose response curve (Fig. 2e). Again, sensitization occurred in Or22a/OrcoWT OSNs with 0.05 µM ethyl hexanoate stimulation (Fig. 5a, b), but not in Or22a/OrcoK339N OSNs with 0.5 µM stimulation (Fig. 5c, d).

### Point mutation in Orco CBS affects odor localization of flies

**Figure 6:**
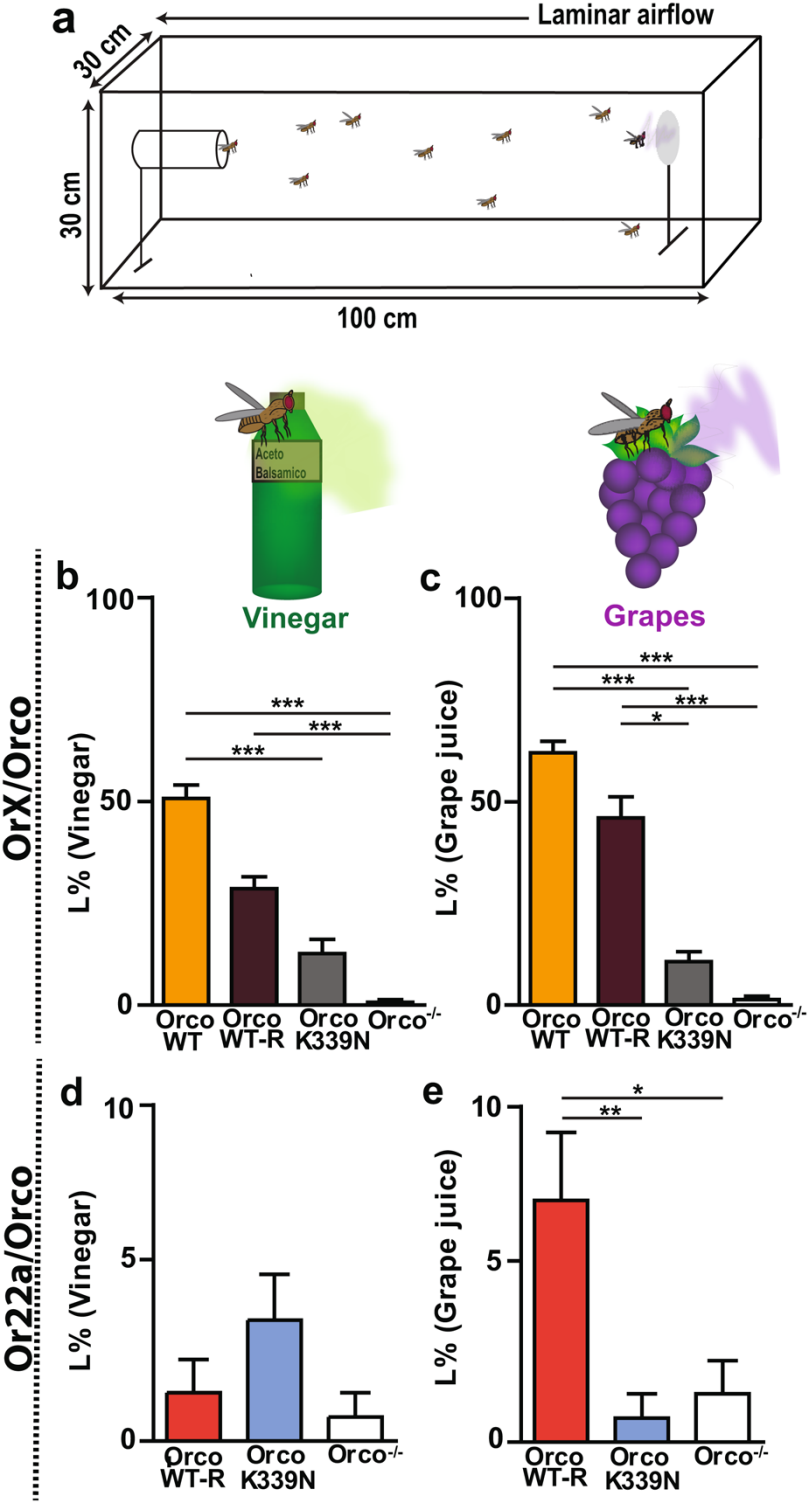
Effect of Orco CBS mutation on the odor odor-guided behaviour. **(a)** Schematic diagram of wind tunnel bioassay. Odor guided behavior is monitored as landing of *Drosophila melanogaster* on filter paper containing balsamic vinegar (10^–2^ dilution) (left) or pure grape juice (right). **(b**, **c)** Landing percentage (L %) of OrX/Orco flies in OrcoWT, OrcoWT-R, OrcoK339N, and Orco^-/-^ condition with vinegar (10^–2^ dilution) **(a)** and pure grape juice **(b)**. **(d**, **e)** Landing percentage (L %) of Or22a/Orco flies in OrcoWT-R, OrcoK339N, and Orco^-/-^ condition with vinegar (10^–2^ dilution) **(d)** and grape juice **(e)**. For **(d)** not all the values were different from each other. n = 15 replicates, each replicate consists of 15 mated female flies. Kruskal–Wallis test with Dunn’s Multiple Comparison Test, *P < 0.05, **P < 0.01, ***P < 0.001, otherwise not significant. Data given as mean ± SEM.

The importance of proper Orco function in long-range odor-guided behavior of *Drosophila* flies has been demonstrated recently^25^. This led to the question whether the OrcoK339N point mutation affects the odor-guided flight performance in the wind tunnel bioassay. We first tested free flight behavior in the wind tunnel bioassay (Fig. 6a) towards the attractant balsamic vinegar^38,39^. We observed that wild type female flies (OrX/OrcoWT), OrX/OrcoWT-R and OrX/OrcoK339N expressing flies forage towards vinegar with immediate upwind flight behaviour. However, while 51% of OrX/OrcoWT and 29% of OrX/OrcoWT-R flies reached the vinegar source, only 12% of OrX/OrcoK339N flies did so (Fig. 6b). To demonstrate the impact of Orco in flight behavior, we tested flies completely lacking Orco (*Orco*^*-/-*^, Orco null mutant). Although *Orco*^-/-^ flies initially oriented towards vinegar, only 1% of them reached the source (Fig. 6b).

Balsamic vinegar is a complex mixture that contains high amounts of acids^38,40^ which may activate certain IRs^41,42^. In a two-choice assay, balsamic vinegar significantly attracted *Orco*^*-/-*^ mutant flies that have only functional IRs (Supplementary Fig. S3). In turn, grape juice, a stimulus attractive to wild type flies, did not attract *Orco*^*-/-*^ null mutant flies (Supplementary Fig. S3), indicating that this alternative attractive stimulus may not activate IRs. To exclude a considerable contribution of IR activation on flight behavior, we additionally tested pure grape juice^40^ as an attractive stimulus in the wind tunnel bioassay (Fig. 6c). We found that 62% of OrX/OrcoWT and 46% of OrX/OrcoWT-R flies reached the grape juice odor source (Fig. 6c). Initially, grape juice also attracted OrX/OrcoK339N flies, but only 10% of them reached the source (Fig. 6c).

We also performed behavioral experiments with flies expressing either wild type Orco (Or22a/OrcoWT-R) or K339N mutant Orco (Or22a/OrcoK339N) solely in Or22a OSNs. However, we found that flies expressing a single functional population of ORs were not sufficiently attracted by vinegar. Only 1% and 7% of Or22a/OrcoWT-R flies reached the vinegar and grape juice sources, respectively (Fig. 6d, e). This prevented any convincing comparison with Or22a/OrcoK339N and *Orco*^-/-^ flies.

## Discussion

We have previously shown that CaM regulates the function of *Drosophila* Orco and OR channels in heterologous expression systems^32,33^. The present study was aimed to test the impact of the point mutation K339N within Orco CBS on the olfactory performance in flies.

We first demonstrated the effect of the OrcoK339N mutation on the primary Ca^2+^ response in OSNs after OR activation. Flies bearing the OrcoK339N mutation in all OrX/Orco-expressing OSNs showed reduced Ca^2+^ responses upon stimulation with the synthetic Orco ligand VUAA1 (Fig. 1). As previously shown, CaM inhibition by W7 reduced Ca^2+^ responses in OSNs^32^. We obtained a qualitatively similar result when we inhibited CaM in OrX/OrcoWT OSNs with W7 (Supplementary Fig. S2). It should, however, be noted that CaM inhibition in cells is not the same event as the manipulation of the CaM binding site in a single protein; in fact, the results were not identical. While the decay time constant of the Ca^2+^ responses in OrX/OrcoK339N became shorter than in OrX/OrcoWT OSNs, the response became prolonged with W7.

In both cases, for OrcoK339N CBS point mutation as well as for CaM inhibition in OX/OrcoWT OSNs, the reduced efficacy in the concentration–response curve was not accompanied by a reduction in sensitivity, i.e. the EC_50_ was not shifted (Fig. 1e and Supplementary Fig. S2b). For heterologously expressed ORs, CaM inhibition had variable effects on Ca^2+^ responses and decay kinetics in different OrX/Orco combinations^32^. For example, while W7 caused a reduced odor response in Or22a/Orco receptors it had no effect in Or33a/Orco complexes. The unchanged sensitivity in our experiments may result from averaging differently affected responses of a variety of OrX/Orco compleses expressed in the OSNs.

Flies expressing solely ORs composed of Or22a/Orco complexes form a drastically reduced system. In this system, the mutation OrcoK339N caused a reduction in OR sensitivity to ethyl hexanoate (Fig. 2e). This result shows that although solely the Orco protein was modified, the complex composed of Or22a and OrcoK339N displayed a change in sensitivity.

When testing Ca^2+^ responses of OrX/Orco expressing OSNs upon stimulation with the ecologically relevant odors vinegar and grape juice that are also preferred stimuli for behavioural experiments, we also noticed reduced responses for OrX/OrcoK339N OSNs in comparison with OrX/OrcoWT OSNs (Fig. 3c, f). This similarity in the effect of vinegar and grape juice on one hand with VUAA1 and ethyl hexanoate on the other one allows to study the impact of the point mutation OrcoK339N on the behaviour of the fly using these mixture compounds as stimuli.

From a previous study we know that Orco can transport to the OSN cilia in the absence of OR proteins and that it is essential for trafficking ORs to OSN cilia^9^. In a recent study, it was shown that severe mutations in the Orco CBS region, such as a single amino acid deletion (Orco^W431∆^), a charge reversal of two positive residues (Orco^RH344EE^), as well as an N and C terminus deletion (Orco^CBS∆^), adversely affected OR trafficking to the dendritic region, and also CaM knock down diminished odorant evoked OSN activity^35^. Interestingly, we found that the point mutated Orco was still able to transport Or22a to the ciliated outer dendrites (Supplementary Fig. S1). This suggests that the effects observed in Or22a/OrcoK339N OSNs sensitivity (Fig. 2e) are due to functional and/or structural changes of the ORs, but not due to limited trafficking of Or22a to the OSN dendrites.

For adjusting the functional properties of an OR, Ca^2+^ signalling plays a central role^21^. One a key regulator of Orco function is protein kinase C (PKC), the function of which relies on the intracellular Ca^2+^ level^22^. There are five PKC phosphorylation sites in the Orco protein that determine the functional properties of Orco, including the sensitivity to cAMP^22,24,25^. Orco mutant proteins in which the serine or threonine residues in the PKC sites were replaced by asparagine showed reduced sensitivity and flies expressing these proteins displayed impaired odor-guided behavior^22,24,25^. Orco was also found to play a critical role in regulating OR function. For example, activation or inhibition of cAMP production or PKC activity enhanced or diminished OR sensitivity, respectively^22,24^. The feedback in this branch is given by Ca^2+^, which facilitates Orco activation, which in turn leads to a higher Ca^2+^ influx.

Furthermore, the aforementioned modulations of enzyme activity also affected the sensitization phenomenon elicited by repeated subthreshold OR activation^24,33^. Inhibition of cAMP production, for example, abolished sensitization in Or22a expressing neurons. Similarly, flies expressing Orco with disrupted PKC phosphorylation sites did not show sensitization^24^.

A second major player in the Ca^2+^ feedback loop is CaM^33^. Activation of CaM improves Orco function leading to more Ca^2+^ influx. Besides, OR sensitization by repeated stimuli was seen to rely on CaM activity. Sensitization of heterologously expressed Orco proteins as well as ORs was prevented in the presence of W7. Similarly, OrcoK339N channels did not show the sensitization phenomenon^33^. In Or22a/Orco expressing OSNs, sensitization obtained with VUAA1 stimulation was also inhibited by W7^33^. However, a partial sensitization was still present in these OSN somata after treatment with W7 when the OR stimulation was performed with ethyl hexanoate. This difference in the impact of CaM inhibition may indicate that the sensitization process in OSNs is a complex phenomenon. To clarify this point, we need to complement previous experiments in heterologous expression systems that allow studying the function of single ORs outside the native cellular environment, with pharmacological experiments in native *Drosophila* OSNs, and our present approach using the OrcoK339N CBS point mutation. Our findings, obtained with this well-defined preparation, demonstrated that sensitization was abolished in cell bodies of OrX/OrcoK339N OSNs (Fig. 4c, d) with threshold concentration of VUAA1. We also specifically tested sensitization in Or22a/Orco OSNs and observed that the Or22a/OrcoK399N mutant ORs (Fig. 5c, d) required a higher ethyl hexanoate concentration to show a response of similar strength as the ORs composed of Or22a/OrcoWT. We found no sensitization in any of Or22a/OrcoK339N OSNs tested. A possible explanation of the difference to the remaining slight sensitization in study with CaM inhibition by W7 might be due to an incomplete CaM inhibition^33^.

Finally, we examined how the CBS point mutation OrcoK339N would affect odor-guided behavior in a long-range attraction paradigm. Insects usually navigate along the food odor plume in an upwind direction^44^. Balsamic vinegar is a strong stimulus that mediates long-range flight attraction in *D. melanogaster*^38^. As it contains volatiles such as acetic acid, 2-phenyl ethanol, acetoin and others compounds that may also activate IRs^41,42^; here we also used grape juice. Interestingly, we found that OrX/OrcoWT flies were more successful in approaching the grape juice than the vinegar (see Supplementary Fig. S4). This difference might depend on more attractive odor components being present in the grape juice^40^, thereby activating a large number of ORs^45,46^. However, for both attractants, flies expressing OrX/OrcoK339N were significantly less successful in getting to the odor source (Fig. 6b, c). Flies expressing only one receptor type—the food odor receptor Or22a—were unable to approach the odor source (Fig. 6d, e). At least for grape juice, a minority of 7% were successful and again flies with the Or22a/OrcoK339N point mutation had even less success. As previously observed, Orco null mutant flies (*Orco*^*-/-*^*)* never landed on the odor source^25^. This fact makes it less probable that any attraction, even for vinegar, was mediated via IR activation.

Taken together, flies carrying the point mutation OrcoK339N show a significantly impaired odor-guided behaviour. Moreover, the CBS mutation produces a more severe phenotype than the mutation of five PKC phosphorylation sites in Orco^25^. 34% of flies carrying this Orco mutation (Orco PKC mutant) landed on the vinegar plume source. By contrast, in our study only 12% of OrX/OrcoK339N flies reached the source. This illustrates the importance of proper CaM function on Orco for the flight performance in long-range attraction. Besides, we could demonstrate that the contribution from all OrX/Orco OSNs improves the odor localization in flies.

In conclusion, CaM action on the Orco protein plays a vital role in olfactory responses, sensitizing ORs and in odor localization performance of the fly. Further investigations on other OR expressing neurons, e.g. for alarm odors such as Or56a, will show whether the observed strict regulation of OR function is a general feature or rather restricted to food odors.

## Material and methods

### *Drosophila melanogaster* flies

Transgenic and experimental *Drosophila melanogaster* fly lines were bred on conventional cornmeal agar medium under a 12 h light and 12 h dark cycle at 25 °C. All experiments were performed with 4–8 days old female *Drosophila melanogaster* flies.

All experiments with WT *D. melanogaster* flies were carried out using the Canton-S strain (Bloomington stock no. 64349). Experiments with Orco null mutants (*Orco*^*-/-*^*)* were carried out using *Orco*^*1*^ or *Orco*^*2*^ (equivalent) strains^43^. The following *Drosophila* fly lines were used to generate the experimental fly lines for Ca^2+^ imaging and wind tunnel experiments, using the GAL4-UAS system^47^: Orco-GAL4 (Bloomington stock no. 26818), Or22a-GAL4 was donated by Dr L. Vosshall (Rockefeller University), UAS-GCaMP6f (Bloomington stock no. 42747), and UAS-DmOrcoK339N (this study). The K339N point mutation was created by site directed mutagenesis in the CBS (^336^SAIKYWER^344^), localized within the second intracellular loop of Orco protein^32^. To generate UAS-OrcoK339N flies, full-length OrcoK339N was digested from pcDNA3.1(-)OrcoK339N^32^ and subcloned into pUAST^48^ using matching restriction sites. The sequences were confirmed by double strand DNA sequencing (Eurofins MWG operon, Germany). *Drosophila melanogaster* UAS-OrcoK339N transformants were generated at BestGene Inc (USA).

For Ca^2+^ imaging transgenic control experiments, we used OrX/OrcoWT flies with the genotype + ; *UAS-GCaMP6f/CyO; Orco-GAL4/TM6B* and Or22a/OrcoWT flies with the genotype (+ ; *UAS-GCaMP6f/CyO; Or22a-GAL4/TM6B)*. These flies express a genetically encoded Ca^2+^ indicator GCaMP6f^49^ in all OSNs expressing OrX/Orco complexes, or specifically in OSNs expressing Or22a/Orco complexes, by means of the *Orco-Gal4* or *Or22a-GAL4* driver line, respectively. GCaMP6f is based on circularly permutated green fluorescent protein (cpGFP) and CaM^49^, which monitors changes in the free intracellular Ca^2+^ concentration [Ca^2+^]_I_ reporting on OR activity_._ For Ca^2+^ imaging in flies carrying the K339N point mutation we generated OrX/OrcoK339N flies with the genotype (*w/w; UAS-GCaMP6f/UAS-OrcoK339N; Orco-GAL4,Orco*^*1*^*/Orco-GAL4,Orco*^*1*^) and Or22a/OrcoK339N flies with the genotype (*w/w; UAS-GCaMP6f/UAS-OrcoK339N; Or22a-GAL4,Orco*^*1*^*/Or22a-GAL4,Orco*^*1*^). For OrX/OrcoK339N and Or22a/OrcoK339N, *Orco-Gal4* or *Or22a-GAL4* drives the expression of *UAS-OrcoK339N* and *UAS-GCaMP6f* transgenes in the *Orco*^*1*^ null mutant background.

For wind tunnel transgenic controls, we used OrX/OrcoWT-R (Orco^-/-^ null mutant rescued with wild type Orco in all OrX OSNs) flies with the genotype (*w/w; UAS-Orco/UAS-Orco; Orco-GAL4,orco*^*1*^/*Orco-GAL4,orco*^*1*^), OrX/OrcoWT (*Canton-S*) flies, and Or22a/OrcoWT-R (Orco^-/-^ null mutant rescued with wild type Orco solely in Or22a OSNs) flies with the genotype (*w/w; UAS-Orco/UAS-Orco; Or22a-GAL4,orco*^*1*^*/Or22a-GAL4,orco*^*1*^)*.* For wind tunnel experiments with flies carrying the K339N point mutation in the CBS of Orco, we generated OrX/OrcoK339N flies (*w/w; UAS-OrcoK339N/UAS-OrcoK339N; Orco-GAL4,orco*^*1*^/*Orco-GAL4,orco*^*1*^) and Or22a/OrcoK339N (*w/w; UAS-OrcoK339N/UAS-OrcoK339N; Or22a-GAL4,orco*^*1*^*/Or22a-GAL4,orco*^*1*^)*.*

A detailed list of all transgenic flies used in this study can be found in Supplementary Table S1.

This study on the vinegar fly *Drosophila melanogaster* was performed in Germany where the research on invertebrates does not require a permit from a committee approving animal research.

### Chemicals

VUAA1 (2-(4-Ethyl-5-(pyridin-3-yl)-4*H*-1,2,4-triazol-3-ylthio)-*N*-(4-ethylphenyl) acetamide) was synthesized by the group “Mass Spectrometry/Proteomics” of the Max Planck Institute for Chemical Ecology (Jena, Germany). W7 (*N*-(6-Aminohexyl)-5-chlor-1-naphthalinsulfonamid-HCl) was purchased from Tocris bioscience (Wiesbaden-Nordenstadt, Germany). Ethyl hexanoate (99% purity) from Sigma Aldrich (Steinheim, Germany). Vinegar (Aceto balsamico di modena 1.G.P (500 ml)) and pure grape juice (Merlot) were purchased. Chemicals used for Ca^2+^ imaging experiments were dissolved in dimethyl sulfoxide (DMSO) to obtain required stock solutions, except for vinegar and grape juice, which were directly dissolved in *Drosophila* Ringer solution.

### Antenna preparation

Antenna dissection was performed as described in^32^. In brief, female flies were anesthetized on ice and heads were separated. Antennae were excised, fixed in a vertical position on a glass slide/cover slip using a two-component silicon curing medium in 800 µl of *Drosophila* Ringer solution (5 mM HEPES; 130 mM NaCl; 5 mM KCl; 4 mM MgCl_2_ 0.6H_2_O; 2 mM CaCl_2_ and 36 mM sucrose; pH 7.3). The funiculus was sectioned to half of its length and was incubated for 5 min to remove air bubbles.

### Ca^2+^ imaging

Ca^2+^ fluorescence detection in antenna preparation was performed using an Axioskop FS microscope (Carl Zeiss, Jena, Germany). Axioskop FS microscope was connected to a monochromator (Polychrome V, TILL Photonics, Gräfelfing, Germany) equipped with an epifluorescence condenser with a water immersion objective lens (LUMPFL 60xW/IR/0.8; Olympus, Hamburg, Germany). Emitted light was separated and filtered with a 490 nm dichroic mirror and a 515 nm long-pass filter. A cooled CCD camera was controlled by TILLVision 4.5.62 software (TILL Photonics) that recorded fluorescence images of OSNs. GCaMP6f labelled OSN was excited at a 0.2 Hz frequency with an exposition time of 50 ms per cycle with 475 nm light. Imaging protocol comprised 180 cycles with 5 s for 1 cycle that was used for dose response and sensitization experiments. After the experiments, regions of interests (ROI’s) were selected from the background (BKG) using TILL Vision software (Version 4.5.62, TILL Photonics). For each time t, BKG was subtracted from ROI i.e., F_t_ = (ROI-BKG) and data were normalised to base level by averaging it from cycle 30^th^ to cycle 39^th^ (F_0_). The response magnitude was calculated as ΔF/F_0_ expressed in percentage i.e. 100_*_(F_t_)/(F_0_). This procedure was performed as described in^32^.

### Dose response experiments

100 µl of VUAA1 was applied at various concentrations for individual OrX/Orco experiments at the cycle 50. Similarly, 100 µl of ethyl hexanoate was applied at various concentrations for Or22a/Orco experiments at the cycle 50. CaM inhibition on OrcoWT (in all OrX/Orco expressing OSNs) was performed by pipetting 100 µl of 10 µM W7 solution at the cycle 40 and then 100 µl VUAA1 solution was applied at the cycle 50. Likewise, 100 µl of vinegar and grape juice was applied at various concentrations for individual OrX/Orco experiments at the cycle 50. All the dilutions were made in *Drosophila* Ringer solution.

### Sensitization experiments

Antennae were continuously perfused with 1000 µl of *Drosophila* Ringer solution in a perfusion chamber (RC-27, Warner Instruments Inc., and Hamden, CT, USA) during the sensitization experiments. 100 µl of 3 µM VUAA1 were manually applied via pipette at cycle 50 and 65 in OrX/Orco experiments. To perform sensitization in Or22a/OrcoWT OSNs we used 0.05 µM ethyl hexanoate, while for Or22a/OrcoK339N flies we used 0.5 µM ethyl hexanoate after the change in sensitivity of Or22a/OrcoK339N OSNs that we found in (Fig. 2e). All chemicals were diluted in *Drosophila* Ringer solution.

### Wind tunnel assay

The wind tunnel bioassay experiments were performed as described^39^. All experiments were performed in Plexiglas wind tunnel of (30 × 30 × 100 cm) placed in a daylight bioassay chamber (Fig. 5a). The temperature was maintained at 28 °C with a relative humidity of 42% in the chamber. The upwind and downwind end of the wind tunnel was covered with polyamide mesh (pore size 0.5 × 0.5 mm; Sintab, Oxie, Sweden). Laminar air flow was maintained at 0.3 m/s by fan (Fischbach GmbH, Neukirchen, Germany) and air was filtered through activated charcoal (14.5 cm diameter. × 32.5 cm long; Camfil, Trosa, Sweden) throughout the wind tunnel.

4 to 8 days old female flies were anesthetized by brief puffing of carbon dioxide and transferred into new food vial. Few hours later female flies were put in an empty food vial stuffed with moist cotton, and were starved for 18 h at RT conditions. Vials containing flies were kept in the chamber for 1 h before the start of the experiments, to introduce fly into new environmental conditions. Ten flies in a vial were considered as one independent replicate. In total, 15 replicates were performed for each fly line mentioned in material and methods. 100 µl of diluted vinegar (10^–2^) or pure grape juice was pipetted on to a filter paper which was fixed on a 12 cm tall metallic filter paper stand. At the upwind side of the wind tunnel, metallic stand was placed and exactly opposite to it, 70 cm away a fly vial was placed on a stand. Flies were released into the chamber and the number of flies reaching the odor source was counted during ten min^39^ as shown in (Supplementary Fig. S3); the maximum number of flies that landed in an interval of 10 min was quantified. The percentage of flies reaching the odor source was calculated for each replicate, and averaged over 15 replicates. All the behavioral data analysis was performed using Prism 4 software (Graph Pad Software, Inc.; LaJolla, CA, USA).

### Statistical analysis

All the statistical analysis was performed using Prism 4 software (Graph Pad Software, Inc.; LaJolla, CA, USA). Data characteristics are given in material methods and figure legends. Between 5 to 11 antennae were used in Ca^2+^ imaging experiments. Data represents mean ± SEM (standard error of the mean) values. Statistical differences were determined using the two-tailed unpaired or paired *t-*test, or the Kruskal–Wallis test with Dunn’s Multiple Comparison Test.

## Supplementary Information

Supplementary Information.
