# Supplementary material for: Calmodulin regulates the olfactory performance in Drosophila melanogaster
Source: Sci Rep. 2021 Feb 12;11:3747. doi: 10.1038/s41598-021-83296-9 (PMC7881240; doi:10.1038/s41598-021-83296-9)
Supplement: Supplementary file 1 — Supplementary Information. [file 41598_2021_83296_MOESM1_ESM.pdf]

Calmodulin regulates the olfactory performance in *Drosophila melanogaster.*

Kalpana Jain[1†], Sofia Lavista-Llanos[1†], Veit Grabe[1], Bill S Hansson[1*], Dieter Wicher[1*]

Affiliation: [1] Max Planck Institute for Chemical Ecology, Department of Evolutionary Neuroethology, Hans-Knöll-Strasse 8, D-07745 Jena, Germany.

[†] Shared authorship

[*] Shared senior authorship

Correspondence to: Dieter Wicher (dwicher@ice.mpg.de)

Supplementary Information

Supplementary information includes four figures with legends, one table, supplementary material and methods, and references.

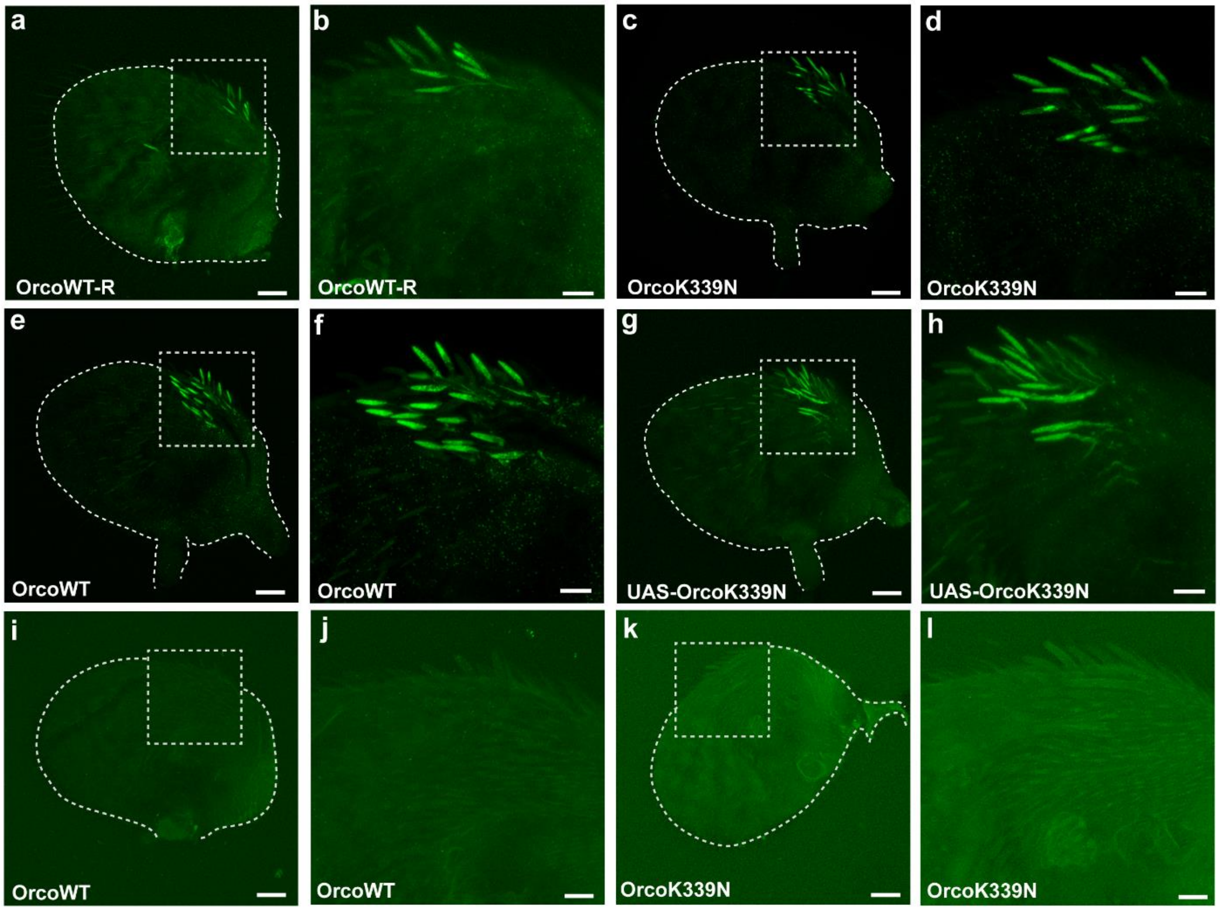

Supplementary Figure S1. Point mutation in Orco CBS does not affect trafficking of Or22a protein in ciliated OSN dendrites. (a-l) Fluorescent images of *Drosophila* antennae stained with Alexa Fluor 488 goat anti- rabbit. Antennae from **a-h** were exposed to Or22a primary antibody, those from **i-l** were not. Antennae marked with white dotted lines are overview projections of OrcoWT-R **(a)**; OrcoK339N **(c)**; OrcoWT **(e)**; UAS-OrcoK339N **(g);** OrcoWT **(i);** OrcoK339N **(k)**. **(b)** Expanded image for OrcoWT-R representing the dotted square in **(a)**, respectively for OrcoK339N **(d)** from **(c)**, OrcoWT **(f)** from **(e)**, UAS-OrcoK339N **(h)** from **(g)**, OrcoWT **(j)** from **(i)**, OrcoK339N **(l)** from **(k)**. Scale bar: overview sections (20 µm), detailed sections (10 µm).

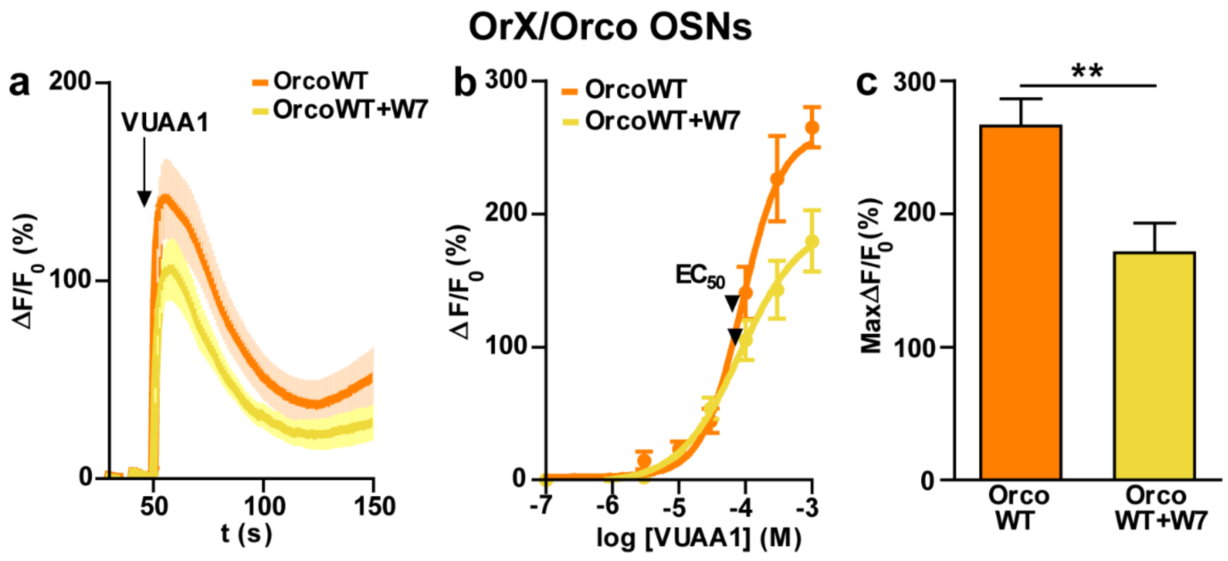

Supplementary Figure S2. Effect of CaM inhibition on the odor response in OrX/Orco expressing OSNs. (a) Time course of Ca^{2+} fluorescence responses of OrX/OrcoWT OSNs under control conditions (OrcoWT) in the presence of 10 µM W7 (OrcoWT+W7) upon stimulation with 100 µM VUAA1 (n = 10 for OrcoWT, n = 8 for OrcoWT+W7). **(b)** Concentration response relationship for conditions as in **(a)** ($7 \leq n \leq 11$). Curves represent sigmoidal fits described by Hill coefficients 1.44 (OrcoWT), 0.94 (OrcoWT+W7), and EC_{50} of 88 µM (OrcoWT), 83 µM (OrcoWT+W7). **(c)** Maximum fluorescence response (Max $\Delta F/F_0$ (%)) obtained with 1 mM VUAA1. n = number of antennae. Two-tailed unpaired *t*- test, **P < 0.01. Data given as mean ± SEM.

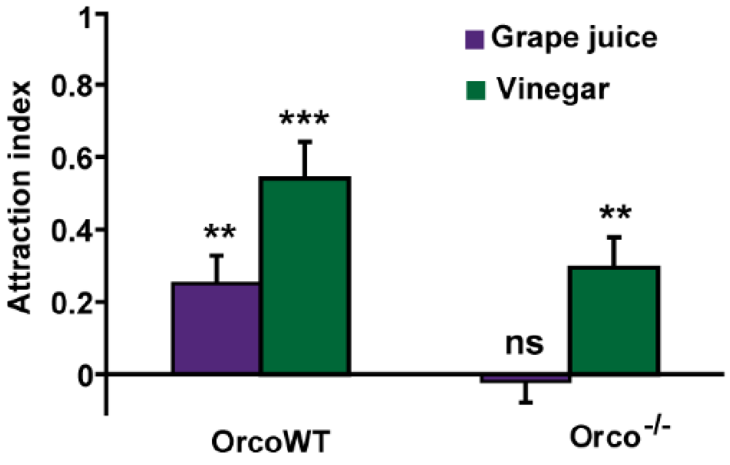

Supplementary Figure S3. Short-range attraction to vinegar and grape juice in the trap assay experiment. Behavioral responses of (OrcoWT, n = 40 for each stimulus) and Orco null-mutants (*Orco*$^{-/-}$, n = 40 for each stimulus) flies to grape juice (10^{-2} diluted in mineral oil, purple) or vinegar (10^{-2} diluted in mineral oil, green) in a trap-assay. n = number of flies.Two-tailed unpaired *t*- test to solvent control (** $P < 0.01$; *** $P < 0.001$); ns = not significant. Data given as mean ± SEM.

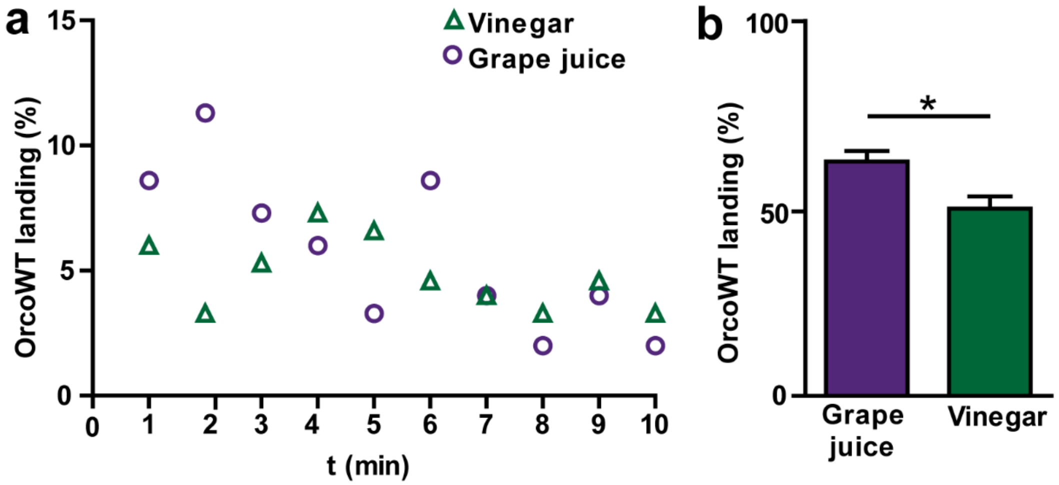

Supplementary Figure S4. Long-range attraction to vinegar and grape juice in the wind tunnel experiment. (a) Landing percentage of OrcoWT flies reaching the odor source (vinegar (green) and grape juice (purple)) as counted within 10 min in wind tunnel bioassay (n = 15). **(b)** Total percentage of OrcoWT flies landing on to the grape juice and vinegar source (n = 15). n = number of flies. Two-tailed unpaired *t*-test, *P < 0.05. Data given as mean ± SEM.

1	Name	Figure(s)	Control	Mutant
Ca^{2+} imaging	**OrX/Orco**	**Figure 1: c, d, e, f**<br>**Figure 3: a-f**<br>**Figure 4: a-d**	**OrcoWT** (+; UAS-GCaMP6f/CyO; Orco-GAL4/TM6B)	**OrcoK339N** (*w/w; UAS-GCaMP6f/UAS-OrcoK339N; Orco-GAL4,Orco*^{1}*/Orco-GAL4,Orco*^{1})
	**Or22a/Orco**	**Figure 2: c, d, e, f**<br>**Figure 5: a-d**	**OrcoWT** (+; *UAS-GCaMP6f/CyO; Or22a-GAL4/TM6B*)	**OrcoK339N** (*w/w; UAS-GCaMP6f/UAS-OrcoK339N; Or22a-GAL4,Orco*^{1}*/Or22a-GAL4,Orco*^{1})
	**OrX/Orco**	**Supplementary Figure S2**	**OrcoWT** (+; *UAS-GCaMP6f/CyO; Orco-GAL4/TM6B*)	**-**
**2**	**Name**	**Figure(s)**	**Control**	**Mutant**
Wind tunnel bioassay	**OrX/Orco**	**Figure 6: a, b**	**OrcoWT** (*Canton-S*)<br>**OrcoWT-R (OrcoWT rescue)** (*w/w; UAS-Orco/UAS-Orco; Orco-GAL4,Orco*^{1}*/Orco-GAL4,Orco*^{1})	**OrcoK339N** (*w/w; UAS-OrcoK339N/UAS-OrcoK339N; Orco-GAL4,Orco*^{1}*/Orco-GAL4,Orco*^{1})<br><br>**Orco**$^{-/-}$ (*Orco*^{1} null mutant)
	**Or22a/Orco**	**Figure 6: c, d**	**OrcoWT-R (OrcoWT rescue)** (*w/w; UAS-Orco/UAS-Orco; Or22a-GAL4,Orco*^{1}*/Or22a-GAL4,Orco*^{1})	**OrcoK339N** (*w/w; UAS-OrcoK339N/UAS-OrcoK339N; Or22a-GAL4,Orco*^{1}*/Or22a-GAL4,Orco*^{1})<br><br>**Orco**$^{-/-}$ (*Orco*^{1} null mutant)
	**OrX/Orco**	**Supplementary Figure S4**	**OrcoWT** (*Canton-S*)	**-**
**3**	**Name**	**Figure**	**Control**	**Mutant**
Trap assay	**OrX/Orco**	**Supplementary Figure S3**	**OrcoWT** (*Canton-S*)	**Orco**$^{-/-}$ (*Orco*^{1} null mutant)
**4**	**Name**	**Figure**	**Control**	**Mutant**
Immunohistochemistry	**anti-Or22a**	**Supplementary Figure S1: a, b, c, d**	**OrcoWT-R (OrcoWT rescue)** (*w/w,; UAS-Orco/UAS-Orco; Orco-GAL4,Orco*^{1}*/Orco-GAL4,Orco*^{1})	**OrcoK339N** (*w/w,; UAS-OrcoK339N/CyO; Orco-GAL4,Orco*^{1}*/Orco-GAL4,Orco*^{1})
		**Supplementary Figure S1: e, f, g, h**	**OrcoWT** (*Canton-S*)<br>**UAS-OrcoK339N (Parental line)** (*w*^{118}*; UAS-DmOrcoK339N/CyO;* +)	**-**
	**no primary antibody**	**Supplementary Figure S1: i, j, k, l**	**OrcoWT** (*Canton-S*)	**OrcoK339N** (*w/w,; UAS-OrcoK339N/CyO; Orco-GAL4,Orco*^{1}*/Orco-GAL4,Orco*^{1})

Supplementary Table S1: Genotype of flies used in this study

Supplementary Material and Methods

Immunohistochemistry

Seven days old mated females from OrX/OrcoWT-R, OrX/OrcoK339N, OrX/OrcoWT (*Canton-S*), and UAS-OrcoK339N were used. Flies were anesthetised on ice and only one antenna (3rd segment) was excised from the head for each replicate. Antennae were fixed with 4% PFA (paraformaldehyde) + 0.1% Triton X-100 (Carl Roth, Germany) for 2 hours on ice, on a rotator. In the next step, antennae were washed with phosphate-buffered saline solution (PBS) containing 0.1 % Triton X-100 (PBST) 3 times for every 20 min. Next, antennae were blocked in blocking solution (normal goat serum (NGS) in PBST) for one hour at room temperature (RT). Afterwards, antennae were incubated with the primary antibody anti-Or22a (1:1000) in blocking solution for 48 hrs at 4˚C. Later, antennae were washed with PBST for four times for 15 min at RT, and then treated with the secondary antibody (Alexa Fluor488 goat anti rabbit IgG (H+L), 1:250 Invitrogen by Thermo Fisher Scientific) in blocking solution for 36 hrs at 4 ˚C. Lastly, antennae were washed again with PBST three times for 20 minutes, and mounted in vectashield (Vector Burlingame, CA, USA). Anti-Or22a was kindly donated by Dr L. Vosshall (Rockefeller University).

Two-choice trap bioassay

We tested the innate attraction of *Drosophila melanogaster* OrX/OrcoWT *(Canton-S)* flies and orco null mutant (*orco*$^{-/-}$) flies to vinegar and grape juice by two-choice trap assays as described in [1]. Shortly, 5 day-old OrX/OrcoWT or Orco$^{-/-}$ flies were starved during 12 hours, with free access to water. Forty samples of twenty five to forty flies each were introduced into a box (10 cm x 8 cm x 10 cm) equipped with two containers (4.5 cm height, 3 cm diameter with a pipette-tip opening of 2 mm), each containing respectively an odor source (grape juice (10^{-2}) or vinegar (10^{-2}) in mineral oil) or solvent (mineral oil). Flies were allowed to choose during 24 hours at 25°C and 75% humidity. An attraction index (AI) was calculated as AI = (O−C) / 30, where O is the number

of flies entering the odorant containing trap and C is the number of flies entered the solvent containing trap. The index ranges from -1 (complete avoidance of the odor source) to 1 (complete attraction to the odor source), whereas a value of 0 characterizes no response, i.e. the odor is not detected, or is neutral. For each stimulus, statistical difference was calculated against 0 (no response) and between genotypes.

Confocal microscopy

Antennae images were captured using cLSM 880 (Carl Zeiss, Oberkochen, Germany) using a 40X water immersion objective (C-Apochromat, NA:1.2, Carl Zeiss). Antennae overview stacks were captured at 1024 x 1024 pixel resolution at 0.49 µm intervals, and for the detailed section at 856 x 856 pixel resolution at 0.49 µm intervals. Fiji Image J software[2] was used to create the stack projections. NA: Numerical aperture. All images used for the figure representation were of 8 bits.

Supplementary References

1 Dweck, H. K. M. *et al.* Olfactory channels associated with the *Drosophila* maxillary palp mediate short- and long-range attraction. *eLife* **5**, e14925, doi:10.7554/eLife.14925 (2016).

2 Schneider, C. A., Rasband, W. S. & Eliceiri, K. W. NIH Image to ImageJ: 25 years of image analysis. *Nature Methods* **9**, 671-675, doi:10.1038/nmeth.2089 (2012).
